# Avascular necrosis of the sesamoid bone of the second metacarpophalangeal joint – a literature review and therapy recommendations

**DOI:** 10.3205/iprs000195

**Published:** 2026-04-24

**Authors:** Antek Nicklas, Adrian Dragu, Kevin Bienger

**Affiliations:** 1 University Center for Orthopedics, Trauma and Plastic Surgery, Department of Plastic and Hand Surgery, University Hospital Carl Gustav Carus at the TU Dresden, Germany

**Keywords:** avascular necrosis, sesamoid bone, hand surgery, therapy recommendations

## Abstract

**Introduction:**

Avascular necrosis of the sesamoid bones in the hand is a very rare and painful condition that is often associated to overuse or circulatory disturbances. The literature on its etiology, cause, and frequency remains sparse. This condition is more commonly observed in the femoral head or metacarpal head, while sesamoid necrosis of the hand is less frequently to not at all discussed. This study explores the causes, symptoms, diagnosis, and treatment options for this rare condition, presenting a case of a 76-year-old woman diagnosed with avascular necrosis of the sesamoid bone in the index finger.

**Material and methods:**

An extensive literature review was conducted using PubMed and Google Scholar, yielding 82 publications from 1990 to 2024. Of these, 80 were excluded due to being related to the foot. A retrospective analysis of the remaining two articles focused on the conservative and surgical treatments for sesamoid necrosis in the hand. The data include demographic information, diagnosis, and treatment outcomes.

**Results:**

Over the past 35 years, only two cases of avascular necrosis of the sesamoid bones in the hand have been documented. Both previous reports involved female patients presenting with symptoms of grip weakness and progressive pain at the metacarpophalangeal (MCP) joint. In the two reported cases, conservative treatments such as Non-steroidal anti-inflammatory drugs (NSAIDs), corticosteroids, and physical therapy were initially applied but ultimately proved ineffective. Surgical intervention, including sesamoidectomy, was required to alleviate symptoms, with all diagnoses confirmed through pathological examination.

**Conclusion:**

Avascular necrosis of the sesamoid bones in the hand, though rare, can lead to significant pain and functional impairment. Conservative treatments can provide short-term symptom relief, but they have been insufficient for long-term pain management or functional restoration in the few cases reported worldwide. Surgical intervention, specifically sesamoidectomy, remains the definitive treatment for achieving lasting relief. Early intervention is recommended to prevent long-term disability, chronic pain and resulting infection.

## Zusammenfassung

**Einleitung:**

Die avaskuläre Nekrose (AVN) der Sesambeine der Hand ist eine sehr seltene und schmerzhafte Erkrankung, die häufig mit Überlastung oder Durchblutungsstörungen assoziiert ist. Die Literatur zu Ätiologie, Ursachen und Häufigkeit ist rar. Diese Erkrankung tritt häufiger am Femurkopf oder an den Köpfchen der Metatarsalen auf, während die Sesambeinnekrose der Hand nur selten bis gar nicht beschrieben wird. Ziel dieser Studie ist es, Ursachen, Symptomatik, Diagnostik und Therapieoptionen dieser seltenen Erkrankung darzustellen sowie einen Fallbericht einer 76-jährigen Patientin mit AVN eines Sesambeins des Zeigefingers zu präsentieren.

**Material und Methoden:**

Es wurde eine umfassende Literaturrecherche in PubMed und Google Scholar durchgeführt, die 82 Publikationen aus den Jahren 1990 bis 2024 identifizierte. Davon wurden 80 Arbeiten ausgeschlossen, da sie sich auf den Fuß bezogen. Die verbleibenden Artikel wurden retrospektiv hinsichtlich konservativer und operativer Therapieansätze der Sesambeinnekrose der Hand analysiert. Erfasst wurden demografische Daten, Diagnosestellung sowie Therapieergebnisse.

**Ergebnisse:**

In den letzten 35 Jahren wurden lediglich zwei Fälle einer AVN der Sesambeine der Hand beschrieben. In beiden Fällen handelte es sich um weibliche Patientinnen, die sich mit verminderter Greifkraft und progredienten Schmerzen im Metakarpophalangealgelenk (MCP) vorstellten. In allen Fällen wurden zunächst konservative Therapien einschließlich nichtsteroidaler Antirheumatika (NSAR), Kortikosteroiden und physiotherapeutischer Maßnahmen durchgeführt, die jedoch letztlich keine ausreichende Wirkung zeigten. Zur Symptomlinderung war eine operative Therapie einschließlich Sesamoidektomie erforderlich. Die Diagnose wurde in allen Fällen histopathologisch gesichert.

**Schlussfolgerung:**

Die AVN der Sesambeine der Hand ist zwar sehr selten, kann jedoch zu erheblichen Schmerzen und funktionellen Einschränkungen führen. Konservative Therapieansätze können kurzfristig zur Symptomlinderung beitragen, sind jedoch entsprechend der verfügbaren Literatur nicht ausreichend für eine dauerhafte Schmerzreduktion und Funktionswiederherstellung. Die operative Therapie im Sinne der Sesamoidektomie stellt die definitive Behandlungsoption dar. Eine frühzeitige Intervention wird empfohlen, um Langzeitschäden, chronische Schmerzen und daraus resultierende Komplikationen zu vermeiden.

## Introduction

The avascular bone necrosis of the sesamoid bone in the hand is a rare but painful condition that often occurs due to overuse or circulatory disturbances. There is little known in the current literature about its etiology, cause, and frequency. Avascular necrosis typically affects the distal end of long bones, particularly the femoral head, and rarely the metacarpal head [1]. The exact cause of avascular necrosis is unclear. It is generally believed that the condition results from inadequate blood supply to the bone [2], [3]. 

This article explores the causes, symptoms, diagnosis, and possible treatment options for this rare condition of the sesamoid bones in the hand. We also present the case of a 76-year-old woman with avascular bone necrosis of the sesamoid bone of the index finger, which initially underwent conservative antibiotic therapy due to infection but developed lytic changes, resulting in the indication for surgical intervention. Known pathologies of the sesamoid bones include fractures, blockages, and sesamoiditis [4], [5], [6]. Wood extensively studied the anatomy, frequency, and pathology of these small bones and reported an incidence of 40–60% for the sesamoid bone of the index finger [7]. Lang and Lourie were able to identify a sesamoid bone in the index finger in approximately 50% of cases; it occurred bilaterally in 84% of cases [8].

In general, sesamoid bones reduce the tension in muscles and tendons by redistributing the forces across the entire muscle or tendon [6].

### Etiology

Avascular bone necrosis of the sesamoid bone is likely caused by reduced blood circulation, leading to bone tissue death. Contributing factors vary by anatomical location:

- Chronic mechanical stress: Particularly relevant for sesamoid bones and other small bones of the foot and hand, where repetitive strain from sports such as tennis, golf, or climbing can predispose to necrosis.
- Traumatic events: Falls or direct impacts can trigger necrosis in both sesamoid and long bones.
- Vascular causes: Systemic conditions such as diabetes mellitus or arterial circulatory disorders are more strongly associated with necrosis of long bones, including the femoral head.
- Corticosteroid therapy: Long-term corticosteroid use increases the risk of osteonecrosis, especially in larger bones such as the femoral head, humeral head, and other long bones.

### Symptoms

The symptoms often develop gradually and may include pain in the area of the base of the index finger or the palm of the hand, particularly during movement or when pressure is applied. Local swelling and warmth may also occur. Additionally, the ability to grip may be significantly impaired.

### Differential diagnoses

When diagnosing avascular bone necrosis of the sesamoid bone, several differential diagnoses must be considered in order to rule out other conditions with similar symptoms:

- Osteoarthritis of the joint near the sesamoid bone as part of a degenerative joint disease, presenting with pain and limited range of motion.
- Stenosing tenosynovitis (Trigger finger): Inflammation of the tendon sheath, causing painful limitations in movement.
- Gout or Pseudogout: Deposition of uric acid crystals or calcium pyrophosphate crystals, leading to inflammatory pain in the joints.
- Infectious arthritis: Bacterial or viral infection of the joint, leading to pain, swelling, and limited movement.

### Diagnostic methods

Diagnosis begins with a detailed medical history and a clinical examination, during which the painful areas are palpated and movement restrictions are assessed. Imaging techniques are then used to evaluate the bone structure more precisely. An X-ray may appear normal in the early stages but shows typical bone changes in advanced cases. Magnetic Resonance Imaging (MRI) is the most sensitive method for detecting bone necrosis, as it can reveal early circulatory disturbances. A scintigraphy can also be used to detect blood supply issues in the sesamoid bone and differentiate between active and healed processes.

### Therapy

Treatment depends on the stage of the disease and the patient’s individual pain symptoms. The goal is to alleviate pain, prevent further progression of the bone necrosis, and preserve hand function as much as possible.

In the early stages, conservative therapy may be sufficient to relieve symptoms and avoid surgery. The affected hand should be rested, and activities that cause strain should be reduced to prevent worsening symptoms. Medications such as nonsteroidal anti-inflammatory drugs (NSAIDs) like ibuprofen or diclofenac are used to reduce pain and inflammation. Additionally, targeted physical therapy can help maintain hand mobility and minimize muscle tension. A temporary immobilization with a splint or bandage may also help alleviate strain and support healing. In some cases, extracorporeal shockwave therapy has been used to promote circulation and stimulate bone regeneration, for example in the feet and other sites of avascular necrosis; however, experience in the hand is lacking, and in our case this approach was not considered due to the need to first rule out infection as a differential diagnosis [9], [10].

If conservative measures are insufficient and symptoms persist or worsen, surgical treatment may be necessary. The choice of surgical intervention depends on the extent of the necrosis and the patient’s individual circumstances. A decompression surgery can reduce pressure in the affected sesamoid bone area, improving blood flow. In severe cases, complete removal of the affected sesamoid bone (sesamoidectomy) may be required, especially when there is significant pain and functional impairment.

## Material and method

To gain a deeper understanding of this rare disease, we conducted an extensive literature review using PubMed and Google Scholar. The search terms “avascular necrosis”, “index finger”, and “os sesamoid” identified 82 publications from 1990 to 2024. 80 of these were excluded as they pertained to sesamoid bones in the foot. Additionally, epidemiological data such as age, gender, and the affected finger or side were analyzed.

This study performed a comprehensive statistical analysis using the latest version of IBM SPSS Statistics to evaluate the data collected from all included publications. The retrospective evaluation was conducted in accordance with the Declaration of Helsinki.

### Case presentation

In this study, we present the case of a 76-year-old female patient who presented to our surgical emergency department with an exacerbation of pain in the right palmar MCP-joint of the index finger. The patient denied any history of trauma or injuries to the skin and soft tissue. Laboratory analysis showed no elevation of infection markers, including C-reactive protein (CRP) and leukocytes, nor an increase in uric acid levels suggestive of gout. Additionally, trigger finger, a history of gout, increased mechanical stress, and rheumatoid arthritis were ruled out. 

**Figure 1:**
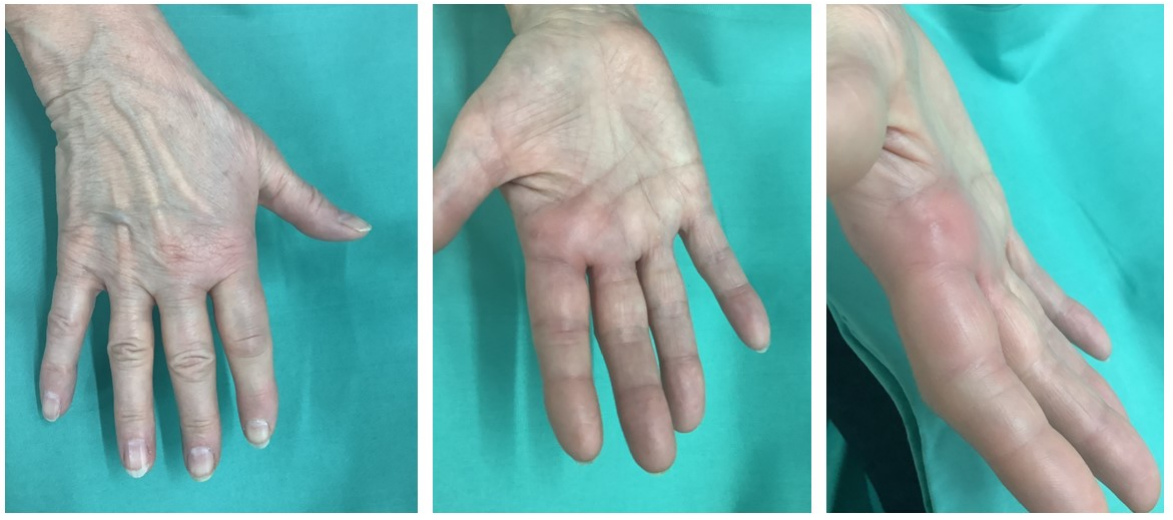
Preoperative soft tissue condition of the 76-year-old patient

Clinically, the patient exhibited localized redness on the palmar aspect of the MCP joint of the index finger, accompanied by significant tenderness on palpation (Figure 1 (Fig. 1)). No triggering phenomenon was observed. Furthermore, Kanavel’s signs, which indicate an infection, were absent [11].

**Figure 2:**
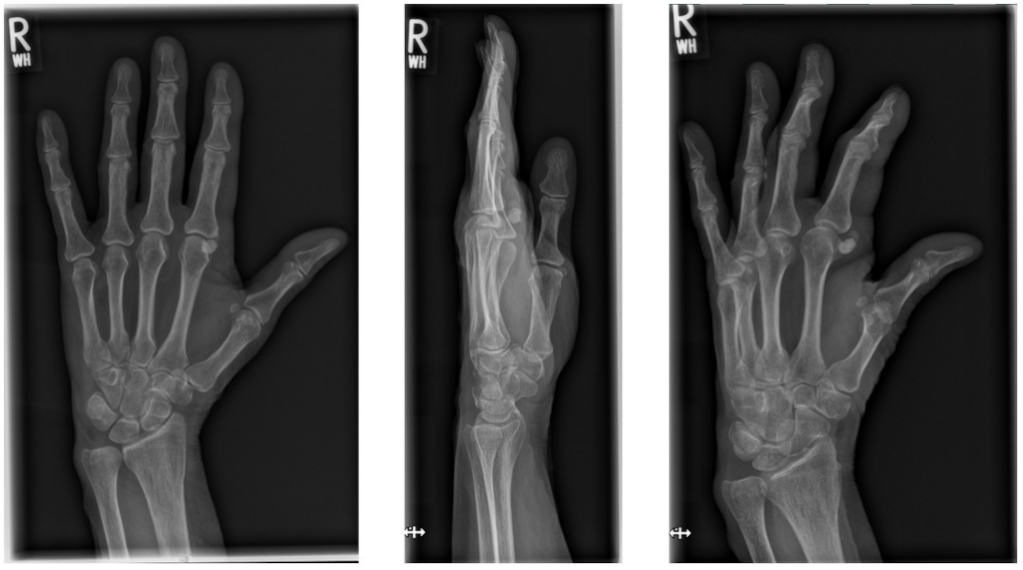
Preoperative X-ray of the right hand in three planes

**Figure 3:**
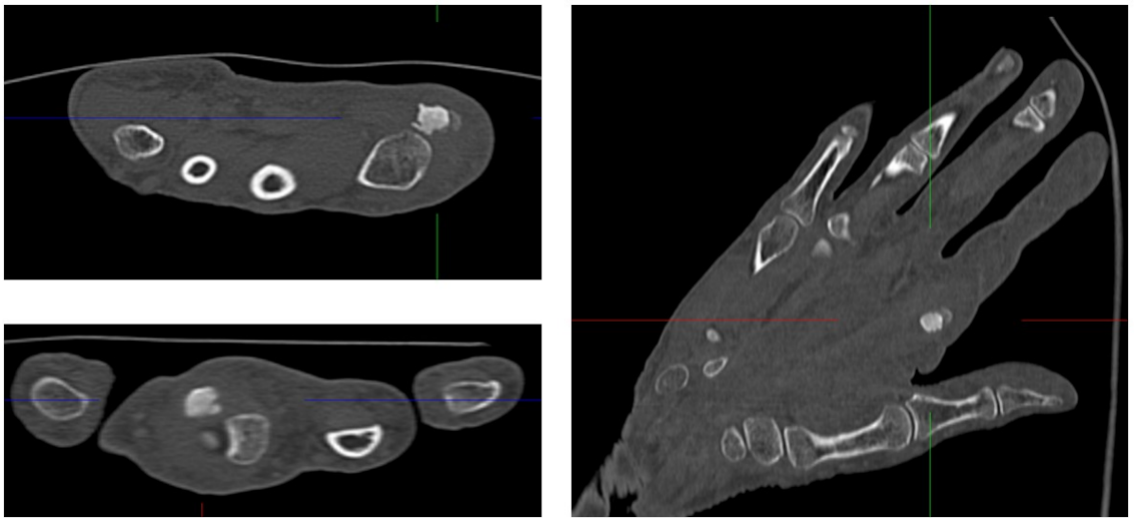
Preoperative Computed Tomography (CT) of the right hand

**Figure 4:**
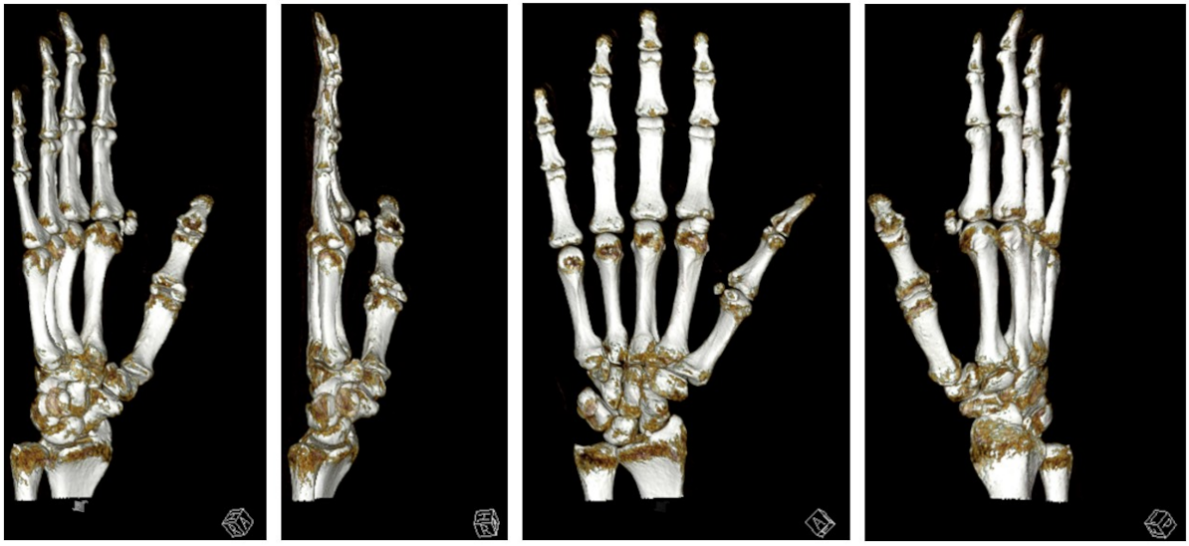
3D reconstruction of sesamoiditis

We performed a radiographic examination of the affected hand, followed by a contrast-enhanced CT scan. Based on the imaging findings, avascular necrosis of the sesamoid bone at the MCP joint was suspected (Figure 2 (Fig. 2), Figure 3 (Fig. 3) and Figure 4 (Fig. 4)).

Given the rarity of this condition, we reviewed the literature and discussed both conservative and surgical treatment options, including sesamoidectomy, with the patient. After an initial 5-day period of immobilization using an intrinsic-plus splint, and in the presence of increasing infection parameters as well as clinical evidence of a spreading infection (positive Kanavel’s sign), we proceeded with surgical intervention in the form of a sesamoidectomy and infection management. Prior to surgery, the patient was treated on an outpatient basis with oral antibiotic therapy (amoxicillin/clavulanic acid, 3 times daily for 5 days).

**Figure 5:**
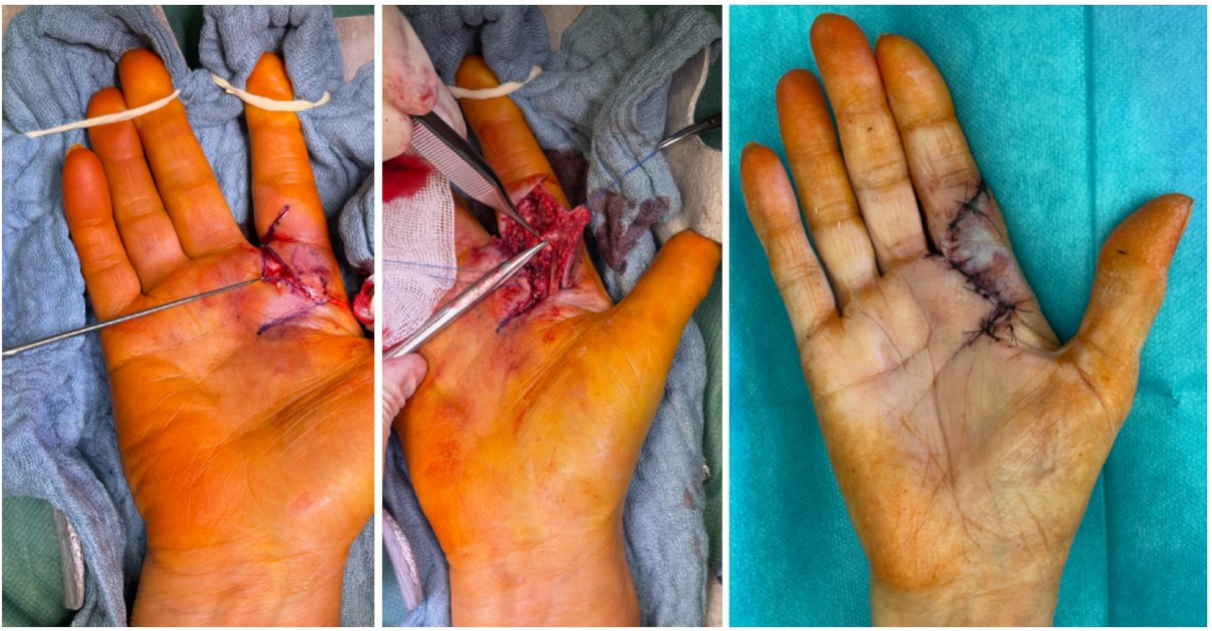
Intraoperative site with visualization of the lytically altered sesamoid bone

The procedure was performed one week after the initial diagnosis under plexus anesthesia (Figure 4 (Fig. 4) and Figure 5 (Fig. 5)). Upon obtaining intraoperative radiographs to plan the surgical approach, the sesamoid bone was no longer radiographically visible. Following the cutaneous incision, we encountered localized sepsis overlying the former sesamoid's location. The bone exhibited extensive osteolysis, with only residual osseous fragments remaining identifiable. A thorough surgical debridement was conducted, encompassing the complete excision of all infected and necrotic tissue (Figure 5 (Fig. 5)). This procedure included the removal of any residual sesamoid fragments, devitalized soft tissue, and purulent material to achieve a clean surgical bed and mitigate the risk of persistent infection. The incision-to-closure time was 84 minutes. Postoperatively, a firm dressing was applied until complete wound healing, and gradual weight-bearing was initiated after 14 days.

The histopathological analysis corresponds to highly fragmented fibroadipose connective tissue with extensive, no longer fresh, florid purulent-necrotizing as well as partly chronic-granulating inflammatory reaction, in places with interspersed avital bone fragments and partially with apparently inflammatorily altered synovial components, overall consistent with an infected lytic avascular bone necrosis. Despite antibiotic therapy, no microbiological pathogen detection was achieved. Consequently, there was no correlation between the histopathological analysis and the lytic transformation of the tissue. Considering all findings, the present case is indicative of avascular necrosis of the sesamoid bone.

Regarding postoperative recovery, the patient reported a significant improvement in pain as early as the second postoperative day. By the time of suture removal on postoperative day 14, the patient was completely pain-free.

## Results

Over the past 35 years, only two cases of avascular necrosis of sesamoid bones in the phalanges of the hand have been described in the literature. These include the publications by Takei in 1996 and van Ash in 2005 [12], [13]. In both case reports, the patients were female, as in our case. All cases, including ours, involved complaints related to the loss of grip strength and progressive pain when pressure was applied to the metacarpophalangeal (MCP) joint area. Differential diagnoses in all cases considered infection, which was ultimately ruled out.

In the known publications, conservative treatments were initially implemented. Van Ash and colleagues treated for six months with NSAIDs and additionally with oral corticosteroid therapy [13], while Takei and colleagues applied corticosteroids locally [12]. However, conservative measures were not ultimately successful in any of the cases and did not lead to adequate symptom reduction or pain relief, so a sesamoidectomy was performed in each case. The diagnosis was confirmed in all cases through pathological examination.

## Discussion

The scientific literature contains numerous articles on the pathology of sesamoid bones, most of which focus on those in the foot, particularly the first metatarsal bone [14], [15]. 

Sesamoid necrosis in the hand is a relatively rare condition, primarily affecting the sesamoid bones, typically located within the tendons, particularly in the thumb. It can lead to chronic pain, swelling, and a loss of function, making effective treatment quite challenging. Our study emphasizes that both conservative and surgical treatment options can be considered for managing this condition. Prompt surgical intervention is indicated in cases of spreading local infection, as described in our case.

In the early stages, conservative therapies like rest, NSAIDs, physical therapy, and splinting can offer temporary symptom relief. These measures help reduce pain and inflammation, allowing patients to continue with their daily activities without significant discomfort. However, the long-term outlook for cases of sesamoid necrosis remains problematic. Our findings suggest that while conservative treatments may slow disease progression or relieve acute symptoms, they typically aren't sufficient to achieve lasting pain relief or full functional restoration. Furthermore, the affected area may be susceptible to secondary localized infection, potentially leading to superimposed septic complications in the compromised tissue.

For cases of advanced or persistent sesamoid necrosis in the hand, surgery remains the most effective treatment approach from our experience and the previous published two case reports. Surgical interventions, such as sesamoid excision, have been shown to significantly reduce pain and improve function, especially when conservative measures fail. This is consistent with the literature, which consistently highlights that removing the necrotic sesamoid is the most reliable way to achieve long-term relief for patients suffering from this condition.

Our analysis confirms that while a conservative approach can be considered initially, particularly in less severe cases, surgical intervention should be recommended for those experiencing ongoing pain or functional impairment. Early surgical intervention generally results in better outcomes, reducing the risk of long-term disability and chronic pain.

In conclusion, while conservative treatments can be useful for the short-term management of sesamoid necrosis in the hand, surgery remains the definitive treatment to achieve lasting pain relief and improve hand function.

Including our study, only three cases of sesamoid bone necrosis in the hand have been reported, highlighting the need for further investigations to establish evidence-based treatment recommendations. Findings from the foot or other anatomical regions cannot be directly extrapolated to the hand, as its unique anatomical and functional characteristics require specific consideration.

## Notes

### Institutional review board statement

The study was conducted in accordance with the Declaration of Helsinki and approved by the Ethics Committee.

### Informed consent statement

Written informed consent for publication has been obtained from the patients, who can be identified, to publish this paper.

### Competing interests

The authors declare that they have no competing interests.
